# CT-guided versus laparoscopic radiofrequency ablation in recurrent small hepatocellular carcinoma against the diaphragmatic dome

**DOI:** 10.1038/srep44583

**Published:** 2017-03-14

**Authors:** Huaiyin Ding, Mu Su, Chuandong Zhu, Lixue Wang, Qin Zheng, Yuan Wan

**Affiliations:** 1 Department of Radiology, The Second Affiliated Hospital of Southeast University, Southeast University, 1-1 Zhongfu Road, Nanjing, Jiangsu, 210003, China; 2 Department of Oncology, The Second Affiliated Hospital of Southeast University, Southeast University, 1-1 Zhongfu Road, Nanjing, Jiangsu, 210003, China; 3 N250, Millennium Science Complex, Pennsylvania State University, University Park, 16801, PA, USA; 4 Nanjing Zetect Biomedical Company, Nanjing, 210003, Jiangsu, China

## Abstract

Computed tomography-guided radiofrequency ablation (CT-RFA) and laparoscopic RFA (L-RFA) have been used to treat intrahepatic recurrent small hepatocellular carcinoma (HCC) against the diaphragmatic dome. However, the therapeutic safety, efficacy, and hospital fee have never been compared between the two techniques due to scarcity of cases. In this retrospective study, 116 patients were divided into two groups with a total of 151 local recurrent HCC lesions abutting the diaphragm. We compared overall survival (OS), local tumor progression (LTP), postoperative complications, and hospital stay and fee between the two groups. Our findings revealed no significant differences in 5-year OS (36.7% *vs.* 44.6%, *p* = 0.4289) or 5-year LTP (73.3% *vs.* 67.9%, *p* = 0.8897) between CT-RFA and L-RFA. The overall hospital stay (2.8 days vs. 4.1 days, *p* < 0.0001) and cost (¥ 19217.6 vs. ¥ 25553.6, *p* < 0.0001) were significantly lower in the CT-RFA in comparison to that of L-RFA. In addition, we elaborated on the choice of percutaneous puncture paths depending on the locations of the HCC nodules and 11-year experience with CT-RFA. In conclusion, CT-RFA is a relatively easy and economic technique for recurrent small HCC abutting the diaphragm, and both CT-RFA and L**-**RFA are effective techniques.

Hepatocellular carcinoma (HCC) accounts for approximately 5% of cancer incidence worldwide, and disease recurrence is the primary cause of treatment failure^1^. Repeated liver resection remains a valid and safe curative therapy option for recurrent HCC; however, due to impaired liver function, multifocal intrahepatic or extrahepatic recurrence, and tumors in unresectable locations, this method is feasible in a minority of patients^2^. In particular, recurrent HCC nodules less than 3 centimeters located in the hepatic dome beneath the diaphragm may represent one of the most difficult sites for resection due to anatomical limitations or more complications/injuries caused by surgery; the morbidity ranges from 16–23% in Chinese^3^,^4^,^5^,^6^,^7^,^8^,^9^,^10^,^11^,^12^,^13^,^14^. To prolong survival and palliate symptoms in patients with HCC, several local, minimally invasive, liver-directed therapies, including transarterial chemoembolization, microwave ablation, radiofrequency ablation (RFA), laser hyperthermia, and other therapies, have been developed in recent decades^15^. Among these techniques, RFA has gained wide acceptance. In RFA, high frequency energy passes through the needle and heats the surrounding tissue, thereby killing nearby cells. RFA for cancer is typically an option when surgery is not possible.

RFA can be performed using percutaneous, laparoscopic or open surgical methods^16^. Minimally invasive surgery is the preferred approach for a growing number of major surgeries that previously involved large incisions and a lengthy recuperation period. Thus, image-guided percutaneous RFA and laparoscopic RFA (L-RFA) are favored^17^. Both approaches have theoretical and proven advantages and disadvantages^18^. Image-guided RFA is convenient, less expensive, and minimally invasive; however, it may encounter shortcomings, such as partial visibility of the tumor, a poor electrode path, and a high risk of collateral thermal damage to the diaphragm^19^. L-RFA decreases the risk of collateral damage, enables the identification of extrahepatic disease, increases the detection sensitivity with intraoperative ultrasound (US), and improves needle placement^20^. However, L-RFA is difficult to perform if patients have extensive intra-abdominal adhesions. Additionally, L-RFA carries the risk of major comorbidities due to general anesthesia, bowel injury, and hepatic decompensation in patients with cirrhosis^21^. These two approaches also show differentiated therapeutic efficacy and safety in HCC. Reports indicate that image-guided RFA leads to a relatively faster recurrence and relatively lower overall survival rate but with lower complication rates^22^. In contrast, L-RFA may provide better curative effects because it enables the surgeon to perform a more aggressive ablation^23^. However, the complication rate of L-RFA is significantly higher than image-guided RFA^24^.

To date, few reports have introduced computed tomography (CT)- or sonography-guided RFA for HCC located in the hepatic dome. In most of the published literature, RFA was performed with artificial pleural effusion and artificial ascites^25^, which pose additional safety concerns including pneumothorax and an increased risk of infection^26^. Moreover, very few studies have established the effectiveness of RFA in the treatment of HCC located in the subphrenic area, assessed therapeutic safety and efficacy, or demonstrated the benefits, including low morbidity, few complications, and repeatability^4^. Notably, fewer than 30 eligible HCC patients were enrolled in each respective study^4^,^5^,^6^,^7^,^8^,^10^,^12^,^13^,^14^, and only two studies focused on intrahepatic recurrent HCC abutting the diaphragm^11^,^14^. To our knowledge, the therapeutic safety and efficacy of image-guided RFA and L-RFA for recurrent small HCC located under the diaphragm have not been compared. In this study, we performed CT-RFA and L-RFA in 60 and 56 patients with recurrent HCC under the diaphragm, respectively. Depending on the tumor location in the subphrenic area, different puncture paths and pretreatment of corresponding intraoperative complications were established. Moreover, we compared postoperative major and minor complications, overall hospital cost, five-year overall survival rate (OS), and rate of five-year local tumor progression (LTP) between CT-RFA and L-RFA for HCC located under the diaphragm within a single institution. Our results indicated no significant difference in OS or LTP between CT-RFA and L-RFA. Regarding postoperative complications, only cases of shoulder and back pain were significantly high in the L-RFA group. Furthermore, the overall hospital cost of L-RFA was significantly higher than that of the CT-RFA group. Thus, CT-RFA is an easy, effective and economic approach for recurrent small HCC abutting the diaphragm.

## Results

### Patients

One hundred and sixteen patients were enrolled in this study. Hepatitis B virus surface antigen was positive in over 80% of patients, while only an average of 2.6% patients showed positive hepatitis C virus antibody. Moreover, over 65% of the patients had liver cirrhosis. Patients with recurrent HCC did not significantly differ in terms of gender, age, Child-Pugh classification, BCLC classification, body mass index (BMI), lineal descent cancer history, alcohol use, HBs Ag (+), Hepatitis C Ab (+), liver cirrhosis, portal hypertension, tumor number, tumor size, or laboratory results between the CT-RFA and L-RFA groups (Table 1). A comparison of recurrent HCC against the diaphragmatic dome revealed no significant differences between the CT-RFA and L-RFA groups.

### Tumor response

Sixty patients with 74 lesions and 56 patients with 77 lesions underwent CT-RFA and L-RFA, respectively. In the CT-RFA group, a mean of 1.35 tumors with a mean diameter of 22.4 mm were treated. The average ablation time was 10.2 ± 1.5 min. After CT-RFA, CT scans were immediately used to evaluate ablation efficacy. Two of the 74 tumors (2.7%) in 2 of the 60 patients (3.3%) were incompletely ablated due to their location, which closely abutted the diaphragm. Patients complained of severe pain during ablation and required ablation abandonment. However, these 2 patients underwent transcatheter arterial chemoembolization (TACE). One month later, the contrast-enhanced CT scans indicated 5 viable residual tumors of 74 tumors (6.8%) in 4 of the 60 patients (6.7%). The patients immediately underwent a second session of percutaneous RFA. In the L-RFA group, a mean of 1.25 tumors with a mean diameter of 23.1 mm were treated. The average ablation time was 9.7 ± 1.6 min. One month after L-RFA, 1 of the 77 tumors (1.3%) in 1 of the 56 patients (1.8%) was observed on the first postoperative CT or MRI scan, which required immediate retreatment with L-RFA of the residual viable tumor areas. Although the local failure rate in the CT-RFA group was higher than that of the L-RFA group one month after ablation, no significant differences were found on a tumor-by tumor basis (*p* = 0.1117) or on a patient-by-patient basis (*p* = 0.3655).

### Complications

No patients died during the ablation treatment. All complications resolved within one week, with the exception of perihepatic abscess. Severe complications, including diaphragmatic perforation, biliary fistula, intra-abdominal hemorrhage, pneumothorax, hemothorax, intestinal perforation, and pleural reaction were not observed. Detailed postoperative complications in the two groups are summarized in Table 2. In the CT-RFA group, transient pleural effusion, transient perihepatic effusion, and liver dysfunction in 6 patients (10%) were detected. In contrast, in addition to these complications, transient heart failure, transient respiratory failure, subcutaneous hematoma, and perihepatic abscess in 7 patients (12.5%) were observed in the L-RFA group. The postoperative minor complications after RFA include hepatalgia, nausea and vomiting, anepithymia, abdominal distension, fever, wound pain, shoulder and back pain, wound infection, and hypercarbia (Table 3). No significant difference was found between two groups in most of above mentioned complications, except should and back pain (Table 2 and 3). Right shoulder pain is an important indicator of diaphragmatic thermal injury or irritation^6^. The number of patients with shoulder and back pain in the L-RFA group was significantly higher than in the other group (*p* = 0.0379). In the CT-RFA group, of the 60 patients, 7 (11.7%) reported mild or moderate right shoulder pain. The duration ranged between 1 and 8 days. In comparison, in the L-RFA group, 15 patients (26.8%) experienced mild and moderate shoulder and back pain, and the duration ranged between 1 and 13 days.

### Hospital stay and costs

The mean hospital stay for patients who underwent CT-RFA was 2.8 ± 0.8 days; this number was 4.1 ± 1.6 days for patients who underwent L-RFA. The hospital stay for patients who underwent L-RFA was significantly higher than that for patients who underwent CT-RFA (*p* < 0.0001). The increased hospital stay in the L-RFA group was directly related to the management of postoperative complications. Hospital financial data were available for all patients included in this analysis (Table 4). The mean hospital cost was significantly higher for patients who underwent L- RFA than for those who underwent CT-RFA. Moreover, the differences in cost between L-RFA and CT-RFA were significant for nearly all items charged.

### Follow-up and Survival

**Figure 1:**
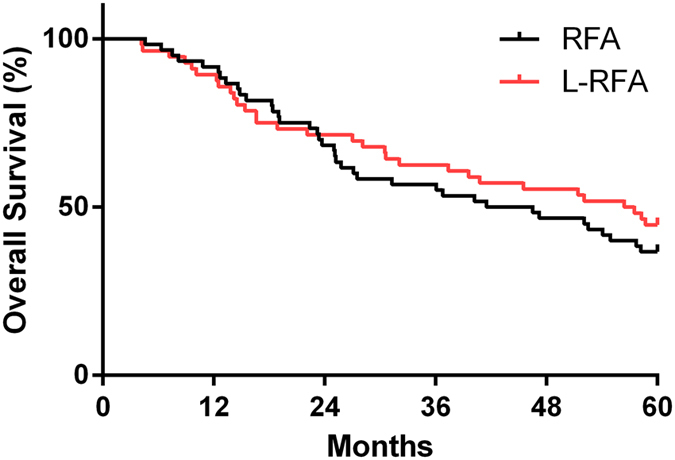
Kaplan-Meier curves of overall survival rates of CT-RFA and L-RFA treated groups of patients with recurrent HCC against the diaphragmatic dome. There was no significant difference between the two groups (*p* = 0.5486).

**Figure 2:**
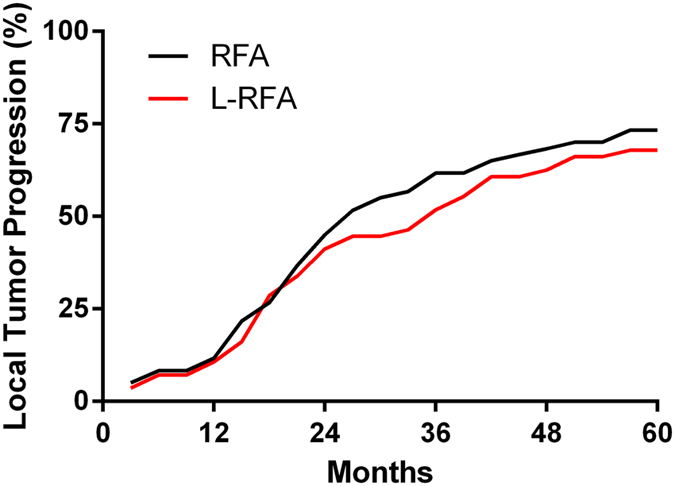
Kaplan-Meier curves of local tumor progression of CT-RFA- and L-RFA-treated groups of patients with recurrent HCC against the diaphragmatic dome. There was no significant difference between the two groups (*p* = 0.5335).

Patients who underwent CT-RFA survived a median of 44 months, and OS at 1, 3, and 5 years was 91.7%, 56.7%, and 36.7%, respectively (Fig. 1). Patients who underwent L-RFA survived a median of 57 months, and OS at 1, 3, and 5 years was 89.2%, 57.1%, and 44.6%, respectively. Our results indicated no statistically significant difference was observed in 5-year OS (*p* = 0.4289) between the two groups. The 1-, 3-, and 5-year local tumor progression rates after CT-RFA and L-RFA were 11.7%, 61.7%, and 73.3%, respectively, in the CT-RFA group and 10.7%, 51.8%, and 67.9%, respectively, in the L-RFA group (Fig. 2). There were no significant differences in 5-year LTP between these two groups as determined using the log-rank test (*p* = 0.8897).

## Discussion

Image-guided RFA and L-RFA are valuable minimally invasive techniques for the ablation of unresectable HCC and demonstrated acceptable morbidity and mortality^27^. Each method has advantages and disadvantages. This study aimed to provide information that would contribute to the creation of evidence-based guidelines for the referral of patients with non-resectable recurrent HCC in the subphrenic area. Both CT and laparoscopic US are more precise in locating tumor lesions and mapping the ablated zone than conventional US, which can be disrupted by the ribs and lungs^28^. In particular, CT-RFA and L-RFA can decrease the risk of severe diaphragmatic injury and/or pneumothorax in the treatment of lesions abutting the diaphragm^29^. Reports have indicated that L-RFA provides better clinical outcomes in terms of survival and complication rates than CT-RFA, laparoscopic hepatectomy, or TACE, which is partially due to the high sensitivity of the detection of small HCC nodules using laparoscopic US and a wider safe margin^30^. However, L-RFA requires general anesthesia; thus, an operator may not be aware of complications (such as diaphragmatic injury from the patient’s response) and may not appropriately manage intraoperative complications. In contrast, CT-RFA generally warrants local sedation. Patients maintain consciousness during ablation and can verbally report pain or discomfort to immediately inform the operator. Admittedly, relying on the patient to report pain as an indicator of diaphragm injury during CT-RFA may be problematic and can lead to unrecognized thermal injury. Moreover, the lesions abutting the diaphragm require immobilization of the liver to permit safe and complete ablation without causing thermal injury to the diaphragm in CT-RFA. Furthermore, CT-RFA faces a challenge in percutaneous puncture, which may not be guided by CT in real time^31^. Thus, the puncture path, angle, and depth must be well managed.

### Immediate local failures, postoperative morbidity and treatment outcomes

Complete ablation has been achieved in a single session in 93% of patients; this is within the expected range (90–98%) according to most image-guided percutaneous RFA studies^32^,^33^,^34^,^35^,^36^,^37^,^38^. The complete ablation rate after initial L-RFA ranges from 90% to 100%^33^,^39^,^40^,^41^. The complete ablation rates of CT-RFA and L-RFA after the first session in this study were similar to those in previous reports. The risk of collateral thermal damage to the diaphragm is known to be relatively higher than that of HCC nodules at other locations, and patients may not experience severe pain during ablation. Due to these difficulties, the ablation of HCC nodules in the subphrenic area may result in a relatively low complete ablation rate and poor clinical outcomes with high morbidity and low local tumor control. However, a high complete ablation rate was achieved in this study, and no patients experienced severe diaphragmatic injury. This finding may be partially due to the puncture and ablation methods established by our group.

The major complications in this study include transient pleural effusion, transient perihepatic effusion, and liver dysfunction. In the current study, the major complication rates were 10% and 12.5% in the CT-RFA group and L-RFA group, respectively. The rate was higher than the average in previous reports. A review indicated that complication rates for percutaneous and L-RFA of hepatic tumors in 3,670 patients were 7.2% and 9.5%, respectively^22^. In another report, an overall complication rate of 7.1% in 2,320 patients who underwent percutaneous RFA at 41 different hospitals was reported^42^,^43^. The major complication rate appeared acceptable considering that all tumors abutted the diaphragm. Minor complications, such as hepatalgia, nausea and vomiting, anepithymia, abdominal distension, fever, wound pain, and shoulder and back pain are common. All patients recovered with or without conservative management. Intraoperative pain or discomfort of CT-RFA for recurrent HCC against the diaphragmatic dome are summarized in Table 5. In the CT-RFA group, shoulder and back pain may be associated with diaphragm injury. The rate (11.7%) was also relatively lower than that of a previous report^12^. In the L-RFA group, the rate of shoulder and back pain was significantly higher than in the CT-RFA group, most likely due to pneumoperitoneum and more aggressive ablation^44^. Additionally, pneumoperitoneum also led to one case of hypercarbia in the L-RFA group^45^.

The inclusion criteria followed in this study led to similar preoperative patient features in the two groups. The present study suggested that CT-RFA and L-RFA resulted in similar survival rates in recurrent small HCC patients with 1–2 nodules. Intraoperative US during L-RFA identified new HCC small nodules in some patients, and a wider safety margin (11 mm) was attained. No statistically significant difference was observed in 5-year OS or 5-year LTP between the two groups; however, we were not able to estimate the effect of the subsequent management of tumor recurrence.

### Hospital stay and cost

A major difference in departmental charges between CT-RFA and L-RFA was observed for anesthesiology, pharmacy, surgical service, surgical unit, and diagnostic imaging. L-RFA is a more expensive approach due to general anesthesia, longer usage of the operating room, laparoscopic surgery, ablation, and increased pain medications after surgery. Additionally, 12.5% of the patients who underwent L-RFA developed complications, which required extended hospital stay, lab tests, and further nursing fees. In contrast, CT-RFA generally required an additional 2–3 plain CT scans, posing extra charges. To monitor the patient’s response in real time, only local anesthesia was performed in this study. The anesthesia-related fee in CT-RFA groups was only approximately ¥25. Our findings are also supported by previous literature. A study by Cassera *et al*. reported that the mean cost of L-RFA for HCC patients was nearly double that of CT-RFA^18^. Importantly, most CT-RFA procedures are performed as outpatient procedures. Due to the limited bed capacity for outpatients and approximately a dozen outpatients for RFA therapy daily, these patients are normally first admitted by the inpatient department. RFA will be performed on the patients on the next day, and the patients will remain in the hospital under observation for up to 24 hours.

Although there is no serious selection bias from patients between two groups, it is a retrospective study with all its inherent defects. Caution is warranted due to limited number of patients. The sample size may have decreased the statistical strength and led to bias. This study can be criticized on the location of lesions. The respective number of lesions in each zone was not recorded. It is more difficult to completely ablate lesions in Zone A in comparison with the rest three zones. Thus, this may introduce the possibility of a selection bias into the data. Moreover, this is a single-center study, and the results may not be generalizable. It is possible that our results may not apply to patients with HCC abutting the diaphragm in other countries because of differences in demographics and underlying causes of liver disease. Further clinical studies, preferably in the form of prospective randomized trials with adequate cases included patients fully followed up, are required to compare the efficacy and safety of CT-RFA and L-RFA in the treatment of recurrent HCC abutting the diaphragm. However, we wanted to obtain some useful information by conducting a retrospective, comparative study before conducting a randomized controlled trial. We are currently conducting a randomized controlled trial to compare between CT-RFA and L-RFA. Larger prospective randomized studies are needed to confirm our results.

In summary, our findings suggest that among patients with recurrent HCC abutting the diaphragmatic dome, no significant differences in OS or LTP were observed between the CT-RFA and L-RFA groups. CT-RFA was less expensive than L-RFA, and patients who underwent CT-RFA experienced a lower rate of postoperative shoulder and back pain. By judiciously determining the puncture path and using ablation methods, close attention must be paid to the patient’s responses, and CT-RFA may be a safe and effective therapy for recurrent small HCC in the subphrenic area.

## Patients and Methods

### Patients

This study was performed according to the guidelines of the Helsinki Declaration. It was registered and approved by the ethics committee at the Second Affiliated Hospital of Southeast University. All patients provided their written informed consent prior to treatment. From January 2009 to March 2012, 116 patients with 151 local recurrent HCC lesions located in the subphrenic dome were treated with either CT-RFA or L-RFA. Data collection was planned before the study. All data used in this study were accurately recorded from the initiation of this study and maintained for analyses. The patients’ characteristics are provided in Table 1. The enrollment criteria were: (1) a diagnosis of HCC confirmed by both histological and cytological examination in all patients; (2) all patients underwent whole-body CT scan to exclude extrahepatic metastases; (3) single nodules ≤3 cm or two nodules with total diameter ≤3 cm; (4) absence of portal vein tumor thrombus or obstinate malignant ascites; (5) Child-Pugh class A or B; (6) prothrombin time ratio >50% (prothrombin time with international normalized ratio <1.7); and (7) a rejection of surgical therapy by the surgeon. The exclusion criteria were: (1) poor or absent visualization of the nodule on CT or US; (2) contraindications for RFA, such as intestinal loops or main bile ducts adjacent to the tumor, severe coagulation disorders (prothrombin activity <40% or platelet <40,000/mL), severe peritoneal adhesion, portal vein embolization, refractory ascites, and distant metastasis; and (3) no foreseeable possibility for liver transplantation.

### Radiofrequency ablation

All RFA procedures were performed on an inpatient basis by H.Y.D. with 11 years’ experience in RFA. All patients fasted overnight. In the CT-RFA group, patients in a supine position received sedation before RFA. After determining the skin entrance point, a single 17-gauge straight electrode was punctured into the liver under normal respiration. Placement of the needle occurred under CT guidance, ensuring that nearby organs were not injured. Subsequent CT scans confirmed the correct orientation of the needle. The patients were treated with Medsphere S-1500 radiofrequency generator (480-KHz) at 40–100 watts with a target temperature of 95 °C for 6 to 12 min. The L-RFA was performed under general anesthesia. The patient was also positioned supine on the operating table. The CO_2_ pneumoperitoneum was established and maintained at approximately 1.3-KPa. Two 11-mm trocars were placed beneath the right costal margin. A diagnostic laparoscopy was used to rule out any extrahepatic disease, and laparoscopic US of the liver was then performed to identify the lesions using a 7.5-MHz 10-mm laparoscopic transducer and US machine. Color flow imaging was performed to assess the vascularity of the lesions. Before ablation, an 18-gauge core needle biopsy of a representative lesion was performed under US guidance using a spring-loaded biopsy gun to confirm the histology. After identifying each lesion, the RFA needle was then placed percutaneously into the lesion. The RFA procedure was similar to that described previously.

### Puncture and ablation strategies

**Figure 3:**
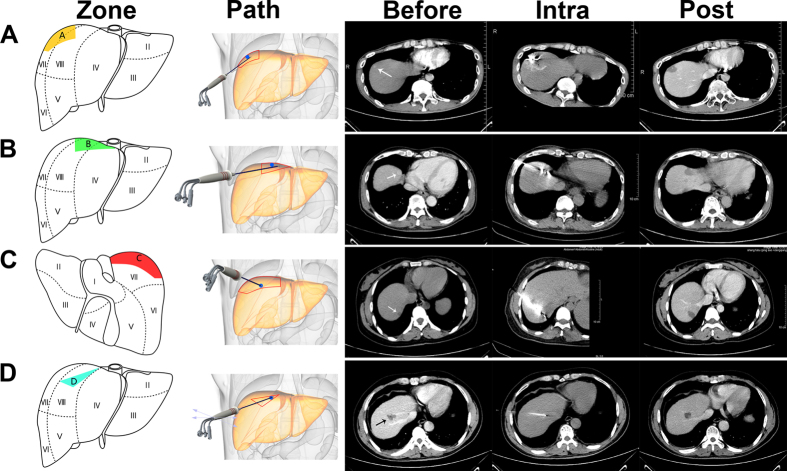
Different puncture paths for ablating nodules at various positions. (**A**) A recurrent small HCC in zone A (arrowhead) was found in a 36-year-old male patient who underwent TACE treatment 8 months previously; no viable tumor was found in the 4-year postoperative CT scan image. (**B**) a recurrent small HCC in zone B (arrowhead) was found in a 61-year-old male patient who underwent hepatectomy 4 years previously; no viable tumor was found in the 2-year postoperative CT scan image. (**C**) a recurrent small HCC in zone C (arrowhead) was found in a 42-year-old female patient who underwent hepatectomy 1 year previously; no viable tumor was found in the 1-year postoperative CT scan image. (**D**) a recurrent small HCC in zone D (arrowhead) was found in a 52-year-old male patient who underwent TACE treatment 3 months previously; no viable tumor was found in the 6-month postoperative CT scan image.

We selected four approaches depending on the location of the lesion (zone A to D) for our puncture and ablation methods (Fig. 3). (i) Nodules in zone A (between the right midaxillary line (RMAL) and right midclavicular line (RMCL)): After determination of the needle puncture site near the RMAL, the needle was pierced upwards slantwise (caudocephalad) at a horizontal angle >45° (Fig. 3A) until the needle tip reached the lower edge of the lesion. To increase the patient’s pain tolerance while ablating small HCC nodules effectively, the needle tip was maintained 30 mm away from the liver capsule. Ablation was performed at 30–40 watts for 30 sec with a 60-sec interval until complete ablation (ablation area: 30 × 22 mm). Right shoulder pain appears frequently during ablation. When the patient complains of shoulder pain or moderate discomfort and the symptom lasts for 20–30 sec, the ablation must be suspended immediately until the patient experiences minimal or no pain. We found that 30 sec was the upper time limit for ablation after pain appearance, and mild to moderate shoulder pain generally disappeared approximately one week after treatment. (ii) Nodules in zone B (between the RMCL and the right cardio-diaphragmatic angle (RCDA)): The needle puncture site was first determined between the RMCL and the RCDA. The needle was pierced upwards slantwise (caudocephalad) with a horizontal angle <45° (Fig. 3B). The needle tip was placed at the edge of the lesion, and puncturing occurred continuously for the following steps. A loose grip of the needle was taken when there was distinguishable tissue resistance. The needle was pushed out 5–10 mm due to movement of the diaphragm; this indicated that the needle tip had reached the diaphragm. The needle tip was maintained in this position and ablation was performed at 30–50 watts for 30 sec with a 60-sec interval until ablation was completed (ablation area: 32 × 24 mm). Ablation of small tumors in zone B commonly causes precordial pain or discomfort. Similarly, ablation was terminated 20–30 sec after pain or discomfort appeared until the patient experienced minimal or no pain. (iii) Nodules in zone C (medial sector of segment VII): After determining the needle puncture site between the RMCL and the RCDA, the needle was slantwise pierced downward (cephalocaudal) (Fig. 3C) until the need tip pierced the lesion and was 5 mm distal from the liver medial capsule. The needle tip was maintained in this position, and ablation was performed at 30 watts for 30 sec with a 60-sec interval until ablation was completed (ablation area: 37 × 15 mm). In the rare case of the nodule closely abutting the capsule, the position of the nodule will move regularly due to respiratory movements. To effectively expand the safety margin to the capsule, we pushed the needle tip forward 5 mm during inspiration and pulled the needle tip back 5 mm during expiration. In contrast with the complications generated in zones A and B during ablation, the common complications were gastrointestinal discomfort and abdominal pain in zone C. Ablation was terminated at 20–30 sec after pain or discomfort appeared until the patient experienced minimal or no discomfort. (iv) Nodules in zone D (between vena hepatica media and vena hepatica dextra): After determining the needle puncture site near the RMCL, the needle was slowly pierced between two veins until the needle pierced the lesion (Fig. 3D). If the patient experienced severe pain or if the operator felt extreme resistance in pushing the needle forward or pulling it back, this may indicate that the needle tip had pierced the veins. Ablation can be performed at 30–50 watts for 2 min with a 60-sec interval (ablation area: 22 × 21 mm). Patients frequently experience gastrointestinal discomfort. Depending on the size and location of the lesion, it may not be completely ablated at one time; an additional session may be required. In all CT-RFA cases, the average safety margin was approximately 8 mm. Most of the lesions closely abutting the diaphragm can be ablated following the above puncture and ablation strategies. The most difficult case, which may warrant an absolute contraindication, is a lesion that is adjacent (less than 5 mm) to intrahepatic vessels.

### Treatment Outcomes and Follow-up

To evaluate local efficacy, contrast-enhanced CT was performed 1 month after the treatment of recurrent HCC in the subphrenic area. Complete ablation was defined as complete non-enhancement of the treated lesion on contrast-enhanced CT. In cases with a viable residual tumor, additional ablation was performed with the aim of complete ablation. If the tumor remained viable after additional ablation, then ablation therapy was considered a failure. Subsequent contrast-enhanced CT scans were repeated every 3 months.

### Statistical Analysis

Statistical analyses were performed using SPSS 16.0 statistical software (SPSS Inc., Chicago, Illinois, USA). All data are reported as the mean ± standard deviation. Significant differences were determined using an independent Student’s t-test, Fisher exact test, or chi-square test. OS and LTP were assessed using the Kaplan-Meier method and compared using the log-rank test. A value of *p* < 0.05 was considered a significant difference.

## Additional Information

**How to cite this article:** Ding, H. *et al*. CT-guided versus laparoscopic radiofrequency ablation in recurrent small hepatocellular carcinoma against the diaphragmatic dome. *Sci. Rep.* **7**, 44583; doi: 10.1038/srep44583 (2017).

**Publisher's note:** Springer Nature remains neutral with regard to jurisdictional claims in published maps and institutional affiliations.

## Figures and Tables

**Table 1:** Comparison of patient groups for CT-RFA and L-RFA for recurrent HCC against the diaphragmatic dome.

*Variable*	*CT-RFA (n = 60) Mean*	*L-RFA (n = 56) Mean*	*Comparison p-value*
*Male/Female*	38/22	40/16	0.3532
*Age*	53.7 ± 6.8	55.2 ± 9.5	0.3278
*Child-Pugh A/B*	35/25	34/22	0.7943
*BCLC A/B*	36/24	37/19	0.4986
*BMI*	22.8 ± 3	23.2 ± 2.9	0.4674
*Lineal Descent Cancer History*	14 (23.3%)	15 (26.7%)	0.6680
*Alcohol Use*	28 (46.7%)	24 (42.9%)	0.6801
*HBs Ag* (+)	50 (83.3%)	46 (82.1%)	0.8648
*Hepatitis C Ab* (+)	2 (3.3%)	1 (1.8%)	0.6000
*Liver Cirrhosis*	41 (68.3%)	35 (62.5%)	0.5091
*Portal Hypertension*	7 (11.7%)	10 (17.9%)	0.3463
*Tumor Number one/two*	46/14	35/21	0.0967
*Mean Tumor Size (mm)*	22.4 ± 3.3	23.1 ± 3.7	0.2838
*Preoperative Lab Results*			
*ALT (IU/L)*	36.5 ± 14.9	30.3 ± 22.6	0.0819
*AST (IU/L)*	35.9 ± 17.0	32.4 ± 12.3	0.2093
*Total Bilirubin (μmol/L)*	21.4 ± 5.4	19.8 ± 6.1	0.1369
*Albumin (g/L)*	38.1 ± 5.8	39.4 ± 3.9	0.1623
*INR*	1.14 ± 0.12	1.16 ± 0.11	0.3524
*Platelet count*(10^9^/L)	138.2 ± 56.5	126.3 ± 69.0	0.3103
*AFP (<25/≥25 ng/mL)*	38/22	43/13	0.1147

**Table 2:** Postoperative major complications after CT-RFA or L-RFA for recurrent HCC against the diaphragmatic dome.

*Characteristic*	*CT-RFA*	*L-RFA*	*Comparison p-value*
*Transient Pleural Effusion*	2	1	1.0000
*Transient Perihepatic Effusion*	3	2	1.0000
*Transient Heart Failure*	0	1	0.4828
*Transient Respiratory Failure*	0	1	0.4828
*Subcutaneous Hematoma*	0	1	0.4828
*Liver Dysfunction*	2	3	0.6714
*Perihepatic Abscess*	0	1	0.4828
*Skin Burn*	0	0	

**Table 3:** Postoperative minor complications after CT-RFA or L-RFA for recurrent HCC against the diaphragmatic dome.

*Characteristic*	*CT-RFA*	*L-RFA*	*Comparison p-value*
*Hepatalgia*			0.3496
*Mild*	18 (30%)	9 (16.1%)	
*Moderate*	10 (16.7%)	13 (23.2%)	
*Severe*	4 (6.7%)	3 (5.4%)	
*Nausea & Vomiting*	23 (38.3%)	23 (41.1%)	0.7629
*Anepithymia*	25 (41.7%)	22 (39.3%)	0.7943
*Abdominal Distension*	18 (30%)	21 (37.5%)	0.3929
*Fever*	26 (43.3%)	27 (48.2%)	0.5980
*Uroschesis*	0	2 (3.6%)	0.2309
*Wound Pain*			0.8648
*Mild*	13 (21.7%)	8 (14.3%)	
*Moderate*	5 (8.3%)	8 (14.3%)	
*Severe*	0	0	
*Wound Infection*	0	1 (1.8%)	0.4828
*Shoulder & Back Pain*			0.0379
*Mild*	5 (8.3%)	11 (19.6%)	
*Moderate*	2 (3.3%)	4 (7.1%)	
*Severe*	0	0	
*Hypercarbia*	0	1 (1.8%)	0.4828

**Table 4:** Overall hospital cost (¥) data for CT-RFA and L-RFA for recurrent HCC against the diaphragmatic dome.

*Department*	*CT-RFA (¥)*	*L-RFA (¥)*	*Comparison p-value*
*Anesthesiology*	25.8 ± 4.5	794.0 ± 64.5	<0.0001
*Pharmacy*	561.2 ± 237.2	1,918.9 ± 474.2	<0.0001
*Surgical Service*	2,502.6 ± 460.3	3,829.7 ± 80.3	<0.0001
*Surgical Unit*	12,621.3 ± 2,236.1	15,654.9 ± 238.3	<0.0001
*Lab Tests*	1,079.3 ± 110.2	1,306.8 ± 266.1	<0.0001
*Diagnostic Imaging*	2,071.1 ± 1,189.6	1,556.1 ± 197.7	0.0018
*Nursing*	118.4 ± 25.0	152.6 ± 43.2	<0.0001
*Ward*	238.7 ± 74.4	340.6 ± 133.7	<0.0001
*Total*	19,217.6 ± 4337.4	25,553.6 ± 1,433.6	<0.0001

**Table 5:** Intraoperative pain or discomfort of CT-RFA for recurrent HCC against the diaphragmatic dome.

HCC Location	Major Symptoms	Degree	Case
Zone A	Shoulder & Back Pain	Mild	12
		Moderate	4
		Severe	0
Zone B	Precordial Pain	Mild	10
		Moderate	2
		Severe	0
Zone C	Gastrointestinal Discomfort* & Abdominal Pain	Mild	16
		Moderate	3
		Severe	0
Zone D	Gastrointestinal Discomfort*	Mild	9
		Moderate	4
		Severe	0

^*^Gastrointestinal discomfort includes a burning sensation, discomfort in the upper abdomen or lower chest, nausea, and belching.
